# Effects of Vitamin D supplementation on physical activity of patients with Heart Failure

**DOI:** 10.12669/pjms.326.10714

**Published:** 2016

**Authors:** Muhammad Zafar Majeed Babar, S.Sabahat Haider, Ghulam Mustafa

**Affiliations:** 1 Muhammad Zafar Majeed Babar, FCPS Medicine. Associate Professor of Medicine, Sheikh Zayed Medical College/Hospital, Rahim Yar Khan, Punjab, Pakistan; 2 S.Sabahat Haider, FCPS Chemical Pathology. (name corrected) Assistant Professor, Senior Demonstrator, Department of Pathology, Sheikh Zayed Medical College/Hospital, Rahim Yar Khan, Punjab, Pakistan; 3 Ghulam Mustafa, MBBS, MSPH. Assistant Professor of Community Medicine, Sheikh Zayed Medical College/Hospital, Rahim Yar Khan, Punjab, Pakistan

**Keywords:** Vitamin D, Congestive Heart failure, dilated cardiomyopathy, Pro-BNP

## Abstract

**Objective:**

To see the role of Vitamin D supplementation on physical status of patients suffering from Congestive Heart Failure (dilated cardiomyopathy).

**Methods:**

In this nonrandomized clinical trial, Forty three Patients with dilated cardiomyopathy who were not showing any significant improvements in physical performance on optimal treatment of heart failure were included. Vitamin D (200,000 IU) supplementation on weekly basis for a period of 12 weeks was added to heart failure treatment. And its effect was seen on 6 minutes’ walk distance and Pro-BNP levels. SPSS version 19 was used for data analysis. Dependent sample t-test was used to see the significant effect of vitamin D supplementation on pre- intervention vitamin D levels, 6MWD and Pro-BNP. Taking p-value <0.05 as significant.

**Results:**

On clinical assessment most of the patients were in NYHA class II (65%), the percentages of NYHA Class I, III and IV was 19%, 9% and 7% respectively. The baseline mean vitamin D level of the study group was 16.59±3.54ng/ml and it raised to 31.97±3.64ng/ml after 12 weeks of supplementation with vitamin D, *p* value<0.0005. The mean distance travelled by the study group before the intervention was 806±380ft while it increased to 945±393ft after the intervention, *p* value of 0.008. The mean of pro-BNP level of the study group before the intervention was 1024±635 while it improved to 159±80 after the intervention with a significant *p* value<0.0005.

**Conclusion:**

Vitamin D supplementation decreases the severity of HF as reflected by reduction in serum pro-BNP levels and significant increase in six minutes’ walk distance.

## INTRODUCTION

Heart failure has now become a progressively more prevailing health issue affecting about 15 million people worldwide and is a major cradle of morbidity and mortality in advanced age patients.^1^,^2^ Despite improvements in understanding the pathophysiology of heart failure, the prognosis is still poor and survival rate is only 35% within five years.^3^,^4^ Vitamin D deficiency is frequent in patients of heart failure, the incidence increases with advancing age.^5^ Vitamin D insufficiency can cause myopathy and muscles weakness and hence reduction in physical activity in patients with and without heart failure.^6^ In addition to calcium and bone homeostasis, vitamin D also possess anti-inflammatory characteristics and can suppress renin concentrations, and increase in muscle strength.^7^-^9^

Main mechanisms by which vitamin D insufficiency can lead to heart failure and other cardiovascular diseases are; Hyperactivity of renin angiotensin aldosterone system, endothelial dysfunction and calcium flux changes leading to reduced cardiac contractility.^10^ Therefore it is hypothesized that vitamin D supplementation in heart failure patients can decrease the progression and severity of heart failure by down-regulating the pre-inflammatory substances, suppressing the renin angiotensin aldosterone system and parathyroid hormone thereby reduction in blood pressure, slowing the myocardial remodeling, by promotion of cell growth and improvements in myocyte contractility.^11^-^15^ There are mixed evidences regarding supplementation of vitamin D in improving the functional outcomes of heart failure patients.^2^,^7^,^16^-^18^

Therefore this study was carried out to see the role of vitamin D supplementation on physical status of patients suffering from Congestive heart failure (dilated cardiomyopathy).

## METHODS

This nonrandomized clinical trial was conducted in department of cardiology, Sheikh Zayed Medical College and Hospital Rahim Yaar khan, Pakistan. The study duration was from 1^st^ Jan 2014 to 30^th^ June 2014. After taking the ethical approval, a total of seventy eight (78) patients were selected, out of which 35 were lost to follow up and 43 patients who continued at the end were examined for study outcomes. Patients having age 15 years to 70 years, diagnosed of having non-ischemic cardiomyopathy (NYHA class I-IV), and vitamin D levels < 30 ng/ml were selected for this study. All these patients were not showing any significant improvements regarding physical activity on optimal heart failure treatment that was being given according to the recent heart failure management guidelines. We just added vitamin D along with the hypertension regimen and examined the patients 12 weeks after adding it. Patients of age less than 15 years, having renal failure or myocardial infarction in the last months, and having valvular heart disease along with cardiomyopathy were excluded from this study. Written informed consent was taken from every patient.

Vitamin D supplementation with 200,000 IU of oral vitamin D supplement was given on weekly basis to every patient for a period of 12 weeks. Serum blood samples for C-reactive proteins, serum calcium, vitamin D levels and pro-BNP were taken before and at the end of treatment. Six minutes’ walk test (6MWT) with monitoring of blood pressure and oxygen saturation was also taken before and after intervention. Distance travelled with time was recorded in every patient.

The severity of HF was assessed by a thorough physical examination. The NYHA function class was assessed in each patient by examining the patients during relaxation, dressing, climbing the stairs and walking.^19^ Six minutes’ walk distance (6MWD) was used to measure the Physical performance of patients according to Guyatt et al. protocol.^20^

Blood samples were taken in the early morning before breakfast in every patient and sent to the laboratory for analysis. Pro-brain natriutetic peptide (Pro-BNP) concentrations were calculated using enzyme immunoassay technique (Siemens Diagnostic Inc, Elkhart, IN).

SPSS version 19 was used for data analysis. Percentages were used for calculation of demographic and NYHA functional class. Dependent sample t-test was used to see the significant effect of vitamin D supplementation on pre-intervention vitamin D levels, 6MWD and Pro-BNP. Taking p-value <0.05 as significant.

**Table-I:** Demographic and NYHA Functional Characteristics.

Variable	Value
Number of patients	43
*Gender*	
Male gender	29 (67.4)
Female gender	14 (32.6)
*Age*	
Mean Age	47.42±11.25
>30 Years	4 (9.3)
30-50 Years	23 (53.5)
>50 Years	16 (37.2)
*Literacy Status*	
Illiterate	38 (88.4)
Literate	5 (11.6)
*Living Area*	
Urban	15 (34.9)
Rural	28 (65.1)
*NYHA Functional Class*	
Class I	8 (18.6)
Class II	28 (65.2)
Class III	4 (9.3)
Class IV	3 (6.9)

**Table-II:** Comparison of Outcome Variables.

Name of Variable	Before Intervention	After Intervention	P-value
Mean Vitamin D levels (ng/mL)	16.59±13.54	31.97±3.64	<0.0005
Distance travelled during 6MWD test (feet)	806±380	945±393	0.008
Pro-BNP levels (pg/mL)	1024±635	159±80	<0.0005

Pro-BNP=Pro Brain Natriuretic Peptide, pg=Pico Gram, 6MWD=6 minutes’ walk Distance.

## RESULTS

Forty three (43) patients were included in the study. Among them 29 (67.4%) were male and 14(32.6%) were female. Four patients (9.3%) were less than 30 years of age, 23 (53.5%) were between the age of 30-50 years. Sixteen (37.2%) were more than 50 years of age. 38(88.4%) patients were illiterate and only five(11.6%) patients were literate. 28(65.1%) belonged to the rural area while 15(34.9%) were from the urban population. On clinical assessment most of the patients were in NYHA class II (65%), the percentages of NYHA Class I, III and IV was 19%, 9% and 7% respectively.

The baseline mean vitamin D level of the study group was 16.59±13.54ng/ml while it was 31.97±3.64ng/ml after the supplementation with vitamin D showing a significant *p* value<0.0005. The mean distance travelled by the study group before the intervention was 806±380ft while it became 945±393ft after the intervention with the significant *p* value of 0.008. The mean of pro-BNP level of the study group before the intervention was 1024±635 while it improved to 159±80 after the intervention with a significant *p* value<0.0005.

## DISCUSSION

In the recent decade, there is tremendous research on the evaluation of vitamin D deficiency in the different populations and its effect on different systems of the body like muscle strength and weakness, brain, prostate, breast and colon tissue and immune cells, which also have vitamin D receptors and are effected by its deficiency. There is a limited research available in the literature which have evaluated the effect of supplementation of vitamin D on functional outcomes of patients suffering from heart failure. Ford et al in their meta-analysis showed that vitamin D therapy can protect the patients against the development of heart failure.^16^

In this study we evaluated the effect of vitamin D after 12 weeks of administration on pro-BNP levels and 6MWD in patients of heart failure. We observed that oral administration of vitamin D3 for 12 weeks in patients of heart failure along with vitamin D deficiency improved their physical level of activity significantly. Previous randomized controlled trial have shown that restoration of vitamin D levels to normal significantly improves muscle contractility and walking capacity and reduces the rate of fall in elderly population.^21^

Amin et al. found that supplementation of vitamin D significantly reduced the heart failure severity and improved the functional activity of patients suffering from heart failure.^2^ In their study they took patients with vitamin D deficiency and without deficiency as well and found that 6MWD increased in all patients and Pro-BNP levels were decreased in their study. Our results were similar to their results we also found that 6MWD was increased significantly after 12 weeks of supplementation with vitamin D. In our study, the six minute walk distance before study was 806±380 feet before surgery and it increased to 945±393 feet after weeks of therapy (p-value 0.008). The pro-BNP levels before study in our study before treatment were 1024±635 pg/mL and it decreased to 159±80 pg/mL after 12 weeks of treatment (P-value <0.0005).

Boxer et al. in their trial did not found any significant difference on 6MWD, oxygen uptake and muscle strength in patients of heart failure after six months of vitamin D supplementation.^17^ Similarly, Witham et al,^18^ also did not found any significant difference of vitamin D on quality of life in patients of heart failure. But these trials recruited only older patients and the risk of muscular disorders in high in this population group. The possible difference in our study and their study outcomes may be due to the age of patients. In our study none of the patient were of >70 years of age. In our study and in the study of Amin et al, young patients were included along with older ones. The possible reason may be the age of study patients that can affect the outcomes of vitamin D supplementation in patients of heart failure.

## CONCLUSION

Vitamin D supplementation decreases the severity of Heart Failure as reflected by reduction in serum pro-BNP levels and significant increase in 6 minutes’ walk distance.
